# Sensor-Assisted Assessment of the Tribological Behavior Patterns of AA7075 Hybrid MMC Reinforced with Multi-Wall Carbon Nanotubes and Pulverized Fuel Ash

**DOI:** 10.3390/ma13112583

**Published:** 2020-06-05

**Authors:** Senthil Kumaran Selvaraj, Kathiravan Srinivasan, Ramesh Kumar S, Yuh-Chung Hu

**Affiliations:** 1 Department of Manufacturing Engineering, School of Mechanical Engineering, Vellore Institute of Technology (VIT), Vellore 632014, India; senthilkumaran.s@vit.ac.in; 2 School of Information Technology and Engineering, Vellore Institute of Technology (VIT), Vellore 632014, India; kathiravan.srinivasan@vit.ac.in; 3 School of Mechanical Engineering, SASTRA Deemed to be University, Thanjavur 613401, India; srk1306@gmail.com; 4 Department of Mechanical and Electromechanical Engineering, National ILan University, No. 1, Sec. 1, Shennong Rd., ILan City 26041, ILan County, Taiwan

**Keywords:** AA7075, multi-wall carbon nanotubes, MMC, material removal rate, pulverized fuel ash, tribology

## Abstract

In recent years, the deployment of sensors and other ancillary technologies has turned out to be vital in the investigation of tribological behavioral patterns of composites. The tribological behavioral patterns of AA7075 hybrid metal matrix composites (MMCs) reinforced with multi-wall carbon nanotubes (MWCNTs), and pulverized fuel ash (PFA) were investigated in this work. The stir casting technique was used to fabricate the composites. The mechanical properties such as tensile strength and hardness were determined for the fabricated material. Besides, microstructure analysis was performed for these AA7075 hybrid MMCs reinforced with MWCNTs and pulverized fuel ash. A pin-on-disc wear testing setup was used to evaluate the wear rate, in which the EN 31 steel disc was used as the counter-face. Taguchi’s design of the experiments was used to optimize the input parameters that impact the characteristics of the hybrid composites, and ANOVA (analysis of variance) was used to determine the contribution of input parameters on the wear behavior. Electrical discharge machining (EDM) was conducted on the AA7075 hybrid metal matrix composites using a copper electrode for determining the material removal rate. These investigations and the results were utilized for determining the optimized output process parameter values of the AA7075 metal matrix composite.

## 1. Introduction

In the present era, composite materials are deployed in various manufacturing sectors, and it plays such a key role in many industries, including aerospace, automobile, bearing, biomedical, defense, and construction firms [1,2,3,4,5]. The carbon nanotubes (CNTs) were considered to be the favorite contenders for improving the characteristics of the composites [6]. The fabrication of the aluminium reinforced with fly ash composites was accomplished through the stir casting process [7,8,9]. Besides, the tribological assessment of these metal matrix composites (MMCs) indicated that there was a linear increase in wear resistance with an upsurge in the fly ash concentration. Furthermore, it was witnessed that the tribological behavior of these composites was influenced by factors such as the applied load, and the size, volume, and nature of the reinforcement particles [9]. Besides, in these composites, it was witnessed that an increase in weight percentage of fly ash causes a corresponding decrease in the wear rate. However, it can also be observed that the uniform distribution of fly ash causes an increase in the hardness of the fabricated composites [10]. The aluminium 7075 hybrid MMCs were prepared through the stir casting process, and the parameters were analyzed using the Taguchi approach [11].

The mechanical properties of Al2024 reinforced with one weight percentage CNTs were investigated [12]. Moreover, the wear behavior of aluminium based synthesized composites were assessed [13,14,15]. Aluminium was reinforced with carbon nanotubes using the induction melting process for aerospace applications [16]. Moreover, it was observed that there was a refinement in crystallite size and augmentation in the lattice strain in the fabricated aluminum/CNTs composites. The reinforcement of AA7075 with Si-Fly ash was investigated, and it was witnessed in the composites that there was a linear increase in tensile strength, hardness, and yield strength when compared to the base alloy [17,18,19]. Al7075 was reinforced with Al_2_O_3_ through the stir casting method, and it was witnessed that the corrosion rate of Al7075 increases with an increase in reinforcement volume. Furthermore, it was noted that Al7075 endures severe corrosion in seawater environments compared to in industrial settings [20]. The tribological and mechanical behavior of Al7075 reinforced with graphite was studied under dry sliding conditions, and it was witnessed that the minimum friction rate at five weight percent of the graphite content [21].

The PFA B_4_C particles were incorporated into the Al7075 matrix by using plasma-activated sintering, and it was observed that the composites exhibited improved mechanical properties with a 7.5 wt.% of B_4_C [22,23]. The mechanical and tribological characteristics of AA7075 reinforced with five weight percentage of TiB_2_ using the ultrasound-assisted casting process, and it was observed that the synthesized material displayed superior wear resistance to the base metal alloy [24,25]. The AA7075 alloy reinforced with alumina through the stir casting process was investigated, and the results showed that synthesized material demonstrated a lower wear rate and reduced coefficient of friction than the base metal matrix [26,27,28]. It can be witnessed from the above articles that creating a high-performance MMC is progressing at a great pace. On the other hand, there is not much focus in the area of combining (in the right proportion) multi-wall carbon nanotubes (MWCNTs) and pulverized fuel ash (PFA) with AA7075 as the base material for creating a new metal matrix composite.

Moreover, the AA7075 is commercially referred to as “aerospace alloy” possessing extraordinary properties such as low weight and high tensile strength. In this work, we have fabricated a hybrid metal matrix composite with AA7075 as the base material that was reinforced with MWCNTs and PFA for creating new metal matrix composite through the stir casting method. It was witnessed that the inclusion of the pulverized fuel ash (PFA) to the base material improved the wear resistance and hardness of the resulting composite material. Moreover, the multi-walled carbon nanotubes were tested to have a tensile strength of 63 GPa, and it possessed a low density of 1.3 to 1.4 g/cm^3^. Further, in house tribological tests revealed that the multi-walled carbon nanotubes are the strongest and stiffest materials concerning factors such as tensile strength and elastic modulus. The strategy of this combination was aimed at developing the AA7075 hybrid MMC that is suitable for aerospace applications. Besides, the assessment of the mechanical properties and wear behavior of this synthesized composite demonstrated supreme results, which will widen its application in other industrial sectors. 

## 2. Materials and Methods

The aluminium alloy 7075-T6 material is characterized by zinc as the primary alloying material with lower compositions of Magnesium, Copper, and few other smaller elements. The base alloy AA7075 was procured from Ultimate Enterprises, Chennai, India, with the standard dimension of (Ø 20 mm × 1000 mm). Multi-walled carbon nano tubes manufactured through the chemical vapor deposition method was obtained with a purity of 99% from Ad Nanotechnologies, Bengaluru, India. The Class F pulverized fuel ash was purchased from Neyveli Lignite Cooperation Ltd, Neyveli, India. 

The crucible and the stirrer were coated with graphite paste to ensure the non-adhesiveness of the molten with them. The furnace heated up to a temperature of 900 °C (above the melting point of aluminum, 670 °C), with the pre-weight AA7075 of 1000 g, converting the solid metal to molten aluminum.

The pre-heated and pre-weighted reinforcements such as MWCNTs and PFA were added to the furnace. Moreover, for every unit percentage increase in temperature, leading to the melting of AA7075, a standard 1% of magnesium was added to the furnace. Furthermore, this process increased the wettability and adhesiveness of the reinforcements with the base material.

The furnace was left for the stirring time of 15 min and stirring speed of 200–300 rpm for the homogenous distribution of reinforcements. The sample with a 10mm diameter and 5 mm in height was taken for the microstructure analysis. The total samples used were five, which is of five different compositions (0, 1, 2, 3, and 4). The polished sample was etched using Keller’s reagent (MilliporeSigma, St. Louis, MO, USA) with the composition: −2 mL HF (48%) + 3 mL HCl + 5 mL HNO_3_ + 190 mL H_2_O. Further, this etchant aided in revealing the grain boundary contrasts and the presence of precipitates in the several synthesized composites. The hardness value was taken for five samples of different compositions (0, 1, 2, 3, and 4). The applied load was 100 kilogram-force (kgf). Then, the indenter was moved 1 mm from the previous spot, and the hardness value was measured. Five values were taken for each sample. The resulting unrecovered indentation diagonals were measured and averaged to give the value in millimeter. The mean values of the five results gave the hardness value of the sample. These length measurements were used to calculate the Vickers Hardness Number (VHN).

The tensile test specimen was prepared as per the ASTM-E8 standards. The fabricated sample had the following dimensions: outer diameter is 15 mm, gauge length is 30 mm, the diameter of the reduced section is 6 mm. The tensile test was accomplished using the Instron machine (Instron, Norwood, MA, USA). In this experiment, only the measurement of the ultimate tensile strength (UTS) is required. The UTS is the maximum amount of stress a material can withstand before failure. Furthermore, this value is the maximum value on the stress–strain graph. By determining the UTS of the material, the maximum strength of the material can be identified.

**Figure 1:**
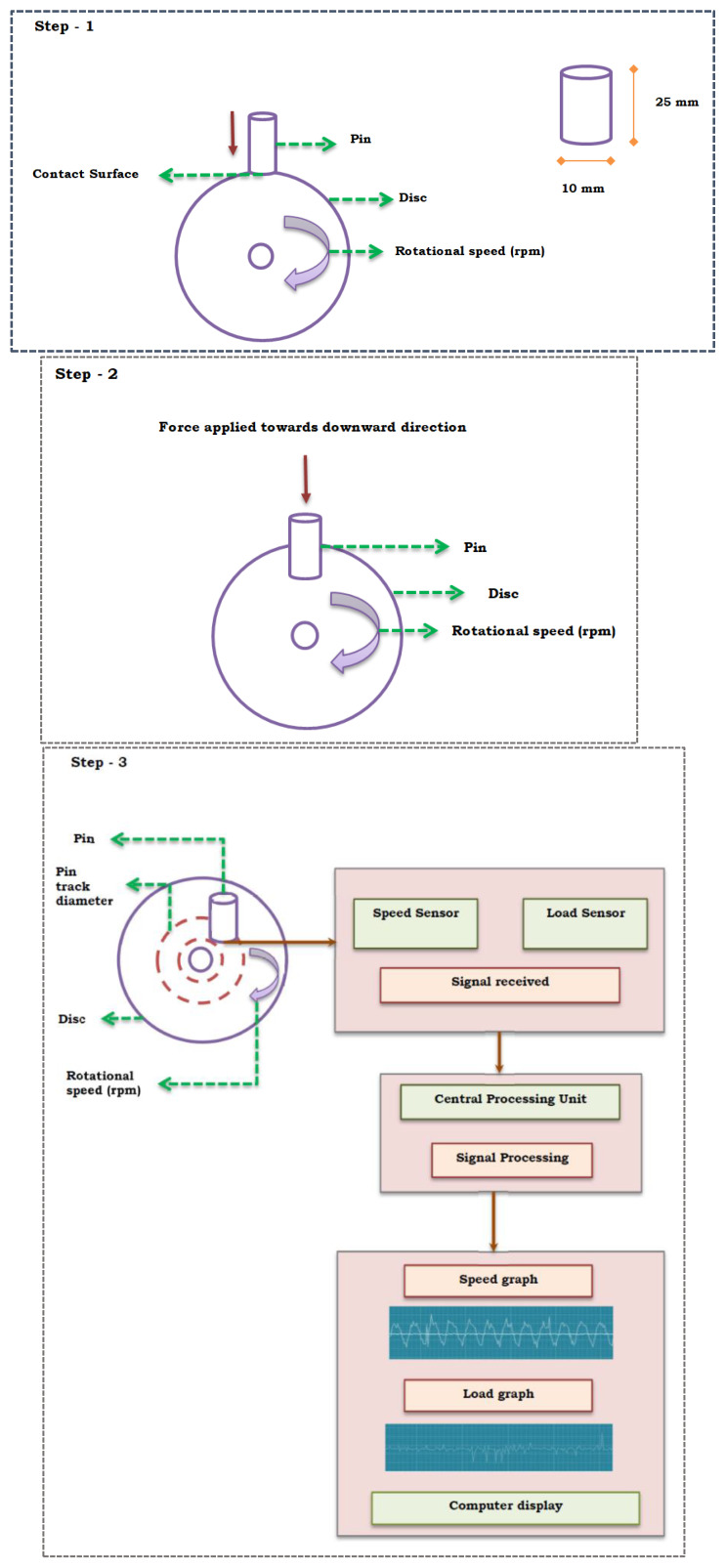
Step 1–3 for speed and load determination using different sensors in the tribology test.

Pin on a disc is a standard procedure for testing the wear rate of the material through the frictional action of the pin over the highly harder disc (EN31 Stainless Steel). Further, this procedure discovers the pre-determined load acting upon the pin. Besides, the procedure accomplished using the pin on the disc setup. The input parameters used for identifying the wear behavior are composites (wt %), load (N), speed (rpm), and time (minutes). Each sample was weighed before and after the procedure to determine the weight loss. The wear rate was also calculated from the obtained results. The setup is shown in Figure 1.

The wire used for this procedure is brass. Usually, the hardness value of the wire is more than that of the material to be cut. The wire is wound over the machine and is arranged accordingly to cut the material. The dimension of the sample is 17 mm diameter and 35 mm height. The sample is held between two clamps and is fixed at both ends. The parameters taken are pulse-on current, pulse-off current, and peak current. The samples must be weighed before and after the procedure to find a weight loss of the sample. With the obtained values, the MRR of the samples is found. 

X-ray powder diffraction (XRD) is a technique used for the identification of the phase of a material. Here the number of samples taken is five. One each for different compositions (0, 1, 2, 3, 4). The range of angle taken is 10° to 90°. The peaks are analyzed between these angles. The dimension of the sample taken is 10 mm diameter and 5 mm height. The analysis was done using the Panalytical X’pert Pro Cu-Ka-band 1.93251 °A (Malvern Panalytical Ltd., Malvern, UK).

## 3. Material Characterization

### 3.1. Details of Experimental Design

The experimental design is most essential to carry out experiments to arrive at a precise output response. Furthermore, this could help us in determining the input variables for various trials to get validated output parameters. In this study, we have used the Taguchi experimental design for determining the wear rate, weight loss, and material removal rate. 

#### Design of Experiments

- Wear Rate and Weight Loss

With the help of MINITAB, we have found that the L_25_ orthogonal array is best suited for our approach of design in determining the wear rate and weight loss with the various sets of factors and levels given below in Table 1.

- Material Removal Rate

Determining the input parameters to find the material removal rate of the developed composites with different levels and factors is fed to MINITAB. Further, this statistical analysis software helps in finding necessary input variables for various trials, as in Table 2. It is also found that L_25_ is best suited orthogonal chart for this experiment.

### 3.2. Constraints

The main constraints that affect the further development of this work are the cost of MWCNTs, and as we all know, the wettability of reinforcements with the base alloy matrix. The previous scenario was due to the different thermal stability of the added reinforcements leading to agglomeration formation. Furthermore, this leads to improper nucleation growth that hinders the mechanical properties such as hardness and tensile strength. Moreover, this could further weaken the strengthening mechanism, such as grain refinement and the dislocation strengthening phenomenon which significantly affect the properties.

Sometimes, the type of PFA and its properties procured may differ with different thermal power plants, leading to deteriorating properties of the developed metal matrix composites. PFA is very poor in dispersion with the base alloy and affects the hardness of the material in a greater context. There is no uniform dimensional stability of the casted product due to shrinkage that happens to base alloy. Besides, the predetermination of allowance is a complicated process as its reinforcements are added to the base alloy, because of there being much less knowledge on theshrinkage properties of the reinforced matrix composites.

## 4. Results and Discussion

**Figure 2:**
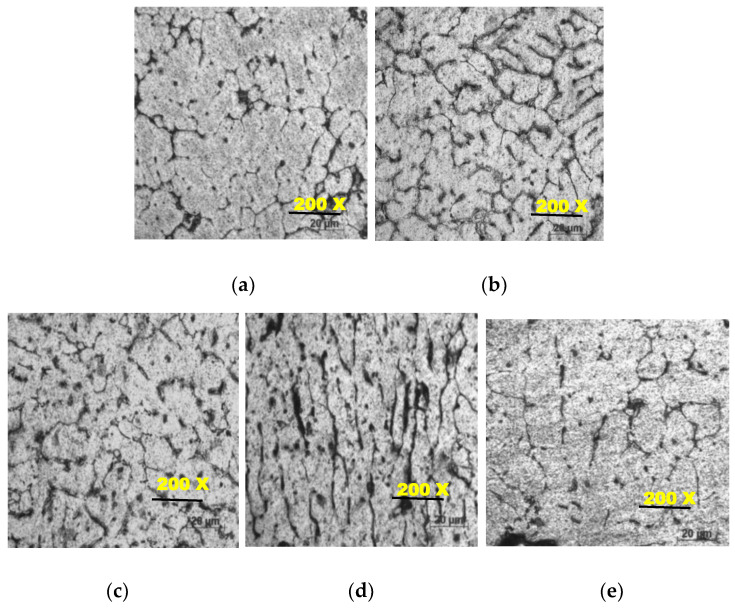
(**a**) Pure AA7075(200×); (**b**) AA7075 + 1%(200×); (**c**) AA7075 + 2%(200×); (**d**) AA7075 + 3%(200×); (**e**) AA7075 + 4%(200×).

The microstructure was taken by an inverted microscope of AA 7075 reinforced with MWCNTs and PFA with various Wt % (0, 1, 2, 3, 4). The microstructure reveals that MWCNT and PFA were mixed and distributed evenly. Figure 2a shows the microstructure of the AA 7075 matrix. Aluminium alloy 7075 particles occupied the primary part, and the rest of the portions were mixed with MWCNTs and PFA particles with irregular shapes, and also the reinforcements were successfully bonded with the matrix material. Figure 2a–e portray the resultant microstructure images from the inverted microscope at the resolution of 200×, which is the best suited and optimal resolution that justifies dispersion of all the incorporated reinforcement such as PFA and multi-wall carbon nanotubes. Furthermore, the uniform distribution of the synthesized composites can be witnessed from the grain boundaries formed in the microstructure images. 

**Figure 3:**
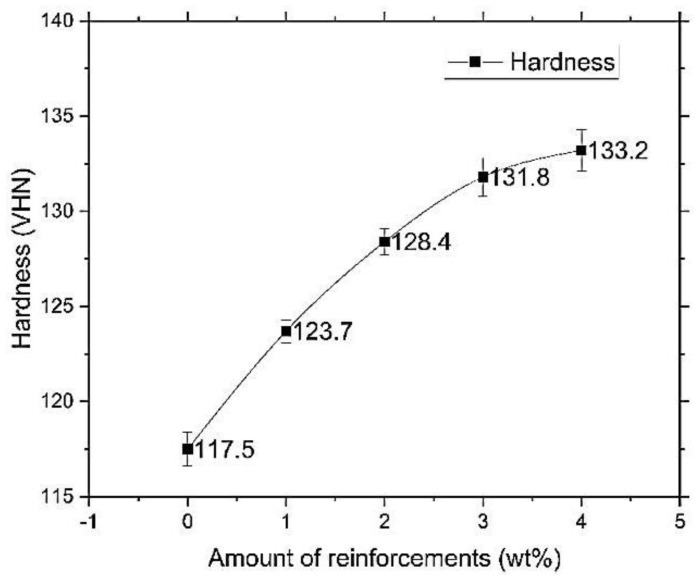
Amount of reinforcements (Wt.%) vs. Hardness (HV).

The phase variations of the baste material that is Aluminium alloy of 7075 with the reinforcements such as MWCNTs and PFA are decipherable. The dispersed phase showing dotted sectors shows the base metal matrix, and the grain boundaries indicate the presence of the reinforcement. However, in our case, one of the reinforcements added is multi-wall carbon nanotubes, which are observed at the nano level, but microscopic studies show that the presence of this reinforcement. The other reinforcement of the hybrid metal matrix is PFA, which is characterized by low density, and volume is mixed with many foreign constituents such as Al_2_O_3_, SiO_2_, at a minimal level. Negligible quantities were infused within the base matrix, which is seen. Due to the high-temperature stir casting process, porosity and cracks can be seen in some parts of the composites. The floating and sinking were reduced in the composite structure because of the reinforcing particles. From this formation, after the solidification, MWCNTs and PFA were retained inside the composites. From Figure 2 of 3% and 4%, it can be seen that the particles are aligned linearly, and increased agglomeration can be seen. The microstructure studies reveal the addition and the successful establishment of the desired levels at the highest possible standards of dispersion in the liquid metal matrix. Figure 3 gives the hardness results for five different compositions (0, 1, 2, 3, 4 ), which were produced by adding MWCNTs and PFA. Figure 3 reveals that the hardness value of AA7075 reinforced with 4% MWCNTs and PFA was 133.2 VH, which was the highest. The hardness value of non-reinforced AA7075 was 117.5 VH while the hardness of AA7075 reinforced with 1% MWCNTs and PFA was 123.7 VH. The hardness value of 2% reinforced MMC was 123.7 VH. The hardness value of 3% reinforced MMC was 131.8 VH, and the hardness value for 4% was 133.2 VH. The hardness value shows a steady rise in increments of the reinforcement at wt.% of unity.

**Figure 4:**
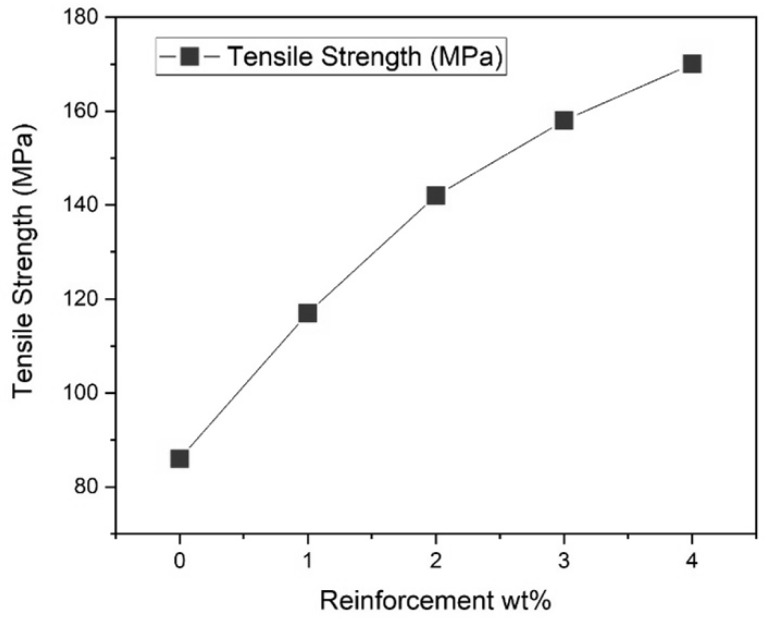
Amount of reinforcements (Wt.%) vs. UTS (MPa).

Figure 4 gives the tensile strength value of five different compositions (0, 1, 2, 3, 4), where the reinforcement particles added are MWCNTs and PFA. Tensile strength is one of the important parameters in mechanical properties. The tensile strength increases with an increase in reinforcement material 4%. The maximum tensile strength is for 4%, where the value is 165 MPa. The ultimate tensile strength for 0%, 1%, 2%, 3%, and 4% are 80 MPa, 112 MPa, 136 MPa,152 Mpa, 165 Mpa respectively. The increase in tensile strength is due to the transmission of stress from the base metal to the reinforcement particles. It shows steady growth in the tensile strength from the non-reinforced base material to reinforced aluminium AA 7075, which indicates that the influence of the reinforcements such as MWCNTs and PFA had a tremendous effect on the tensile force of capability of the samples.

The load capacities from the test that is the ultimate load-bearing capacity of aluminium alloy AA7075 is 1370 N. The load capacities from the test that is the ultimate load-bearing capacity of aluminium alloy 7075 reinforced with 1% of MWCNTs and PFA is 2120 N. The load capacity from the test that is the ultimate load-bearing capacity of aluminium alloy 7075 reinforced with 2% of MWCNTs and PFA is 3330 N. The load capacity from the test that is the ultimate load-bearing capacity of aluminium alloy 7075 reinforced with 3% of MWCNTs and PFA is 4410 N. The load capacity from the test that is the ultimate load-bearing capacity of aluminium alloy 7075 reinforced with % of MWCNTs and PFA is 5120 N. Since both MWCNTs and PFA have brittle characteristics the ductility of the MMC is greatly reduced. With the increase in wt.%, the grain size and homogenous microstructure are more refined, and it leads to superior wear resistance. The force bearing also increases upon the weight percentage of reinforcements in the metal matrix, which is a clear indication of the increase in the desired output.

### 4.1. Wear Behavior

**Figure 5:**
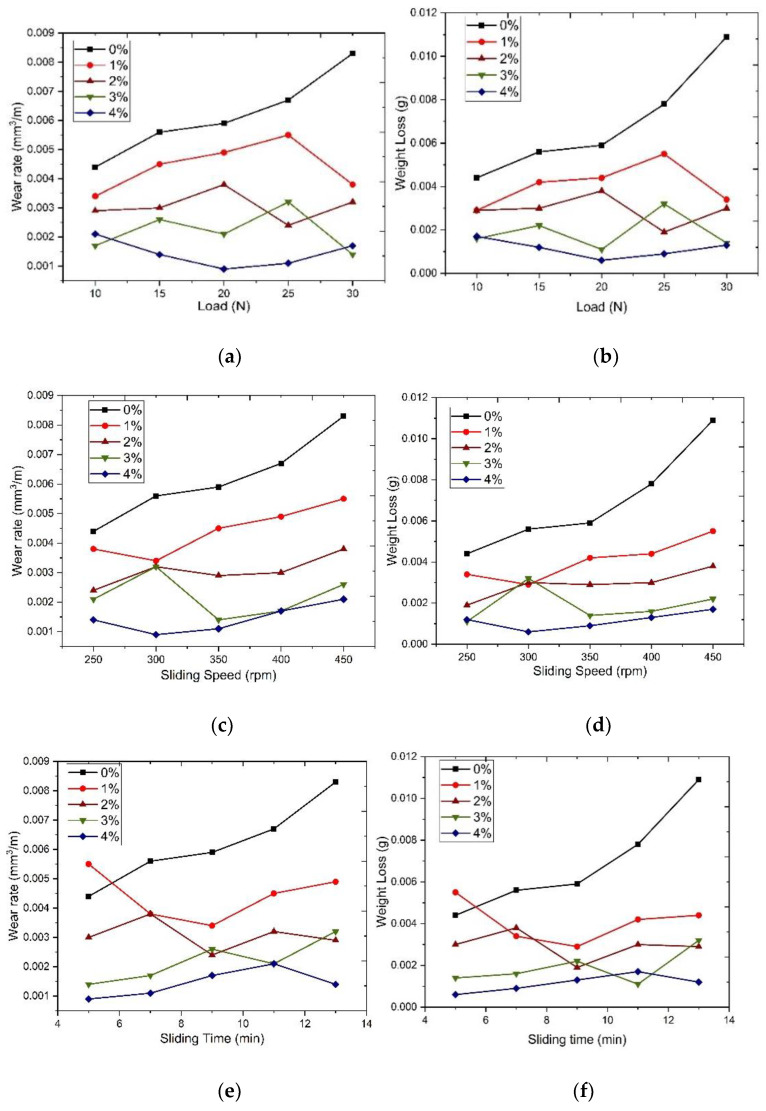
Removal rate and weight loss response according to load, sliding speed and sliding time. (**a**,**b**) can be witnessed that in most cases, the wear rate and weight loss decreases, with a corresponding increase in the composites (wt.%), also taking into account the different values of load (N); (**c**,**d**) can be observed that in most cases, the wear rate and weight loss decreases, with a corresponding increase in the composites (wt.%), also taking into account the different values of sliding speed (rpm); (**e**,**f**) can be perceived that in most cases, the wear rate and weight loss decreases, with a corresponding increase in the composites (wt.%), also taking into account the different values of sliding time (minutes).

Taguchi orthogonal array was chosen to determine the process variables of the input parameters, as Taguchi’s method helps in the optimization of the response parameters concerning concerned factors. We have chosen various weight percentages such as level, and different input parameters such as load (10, 15, 20, 25, 30) N, sliding speed (250, 300, 350, 400, 450) rpm, sliding time (5, 7, 9, 11, 13) min, composites (0%, 1%, 2%, 3%, 4%) as factors. Using Minitab 18, we chose L 25 orthogonal array to find responses such as weight loss and wear rate. Taguchi helps in finding an overall best output parameter, as shown in Table 3, and plots are shown in Figure 5.

**Figure 6:**
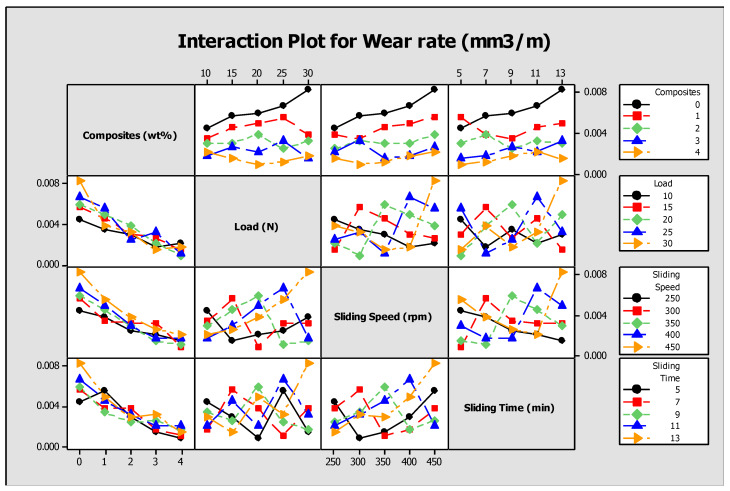
Interaction plot for wear rate.

The analysis of variance is conducted with the Minitab statistical software to quantify the amount of percentage of contribution of each factor on the response, as shown in Table 4. The dominating influencing input process parameter is composites and wear rate and weight loss as response parameters. This marks a line that higher the addition of reinforcements, it owes to lesser wear rate and weight loss, thus by defining our objective. The ANOVA for wear rate and weight loss is shown in Table 5. The full interaction plot shown in Figure 6 illustrates the various influences of input parameters on the output response.

**Figure 7:**
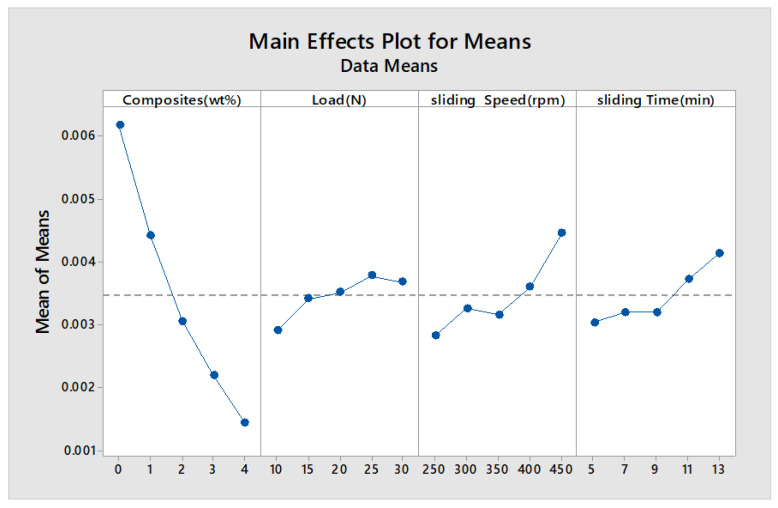
Main effects plot (Wear rate).

**Figure 8:**
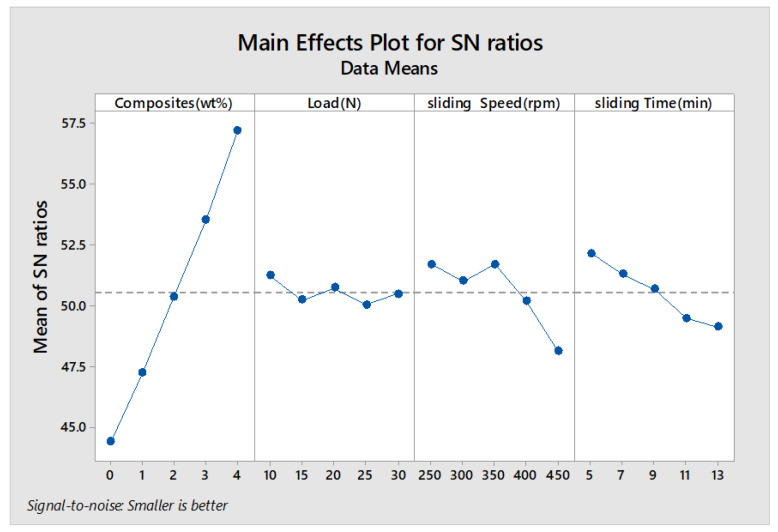
S/N Ratio plot (Wear rate).

**Figure 9:**
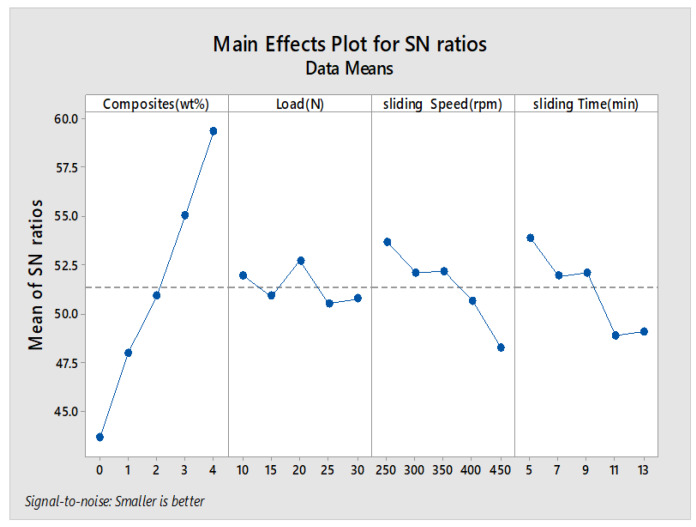
Main effects plot (weight loss).

**Figure 10:**
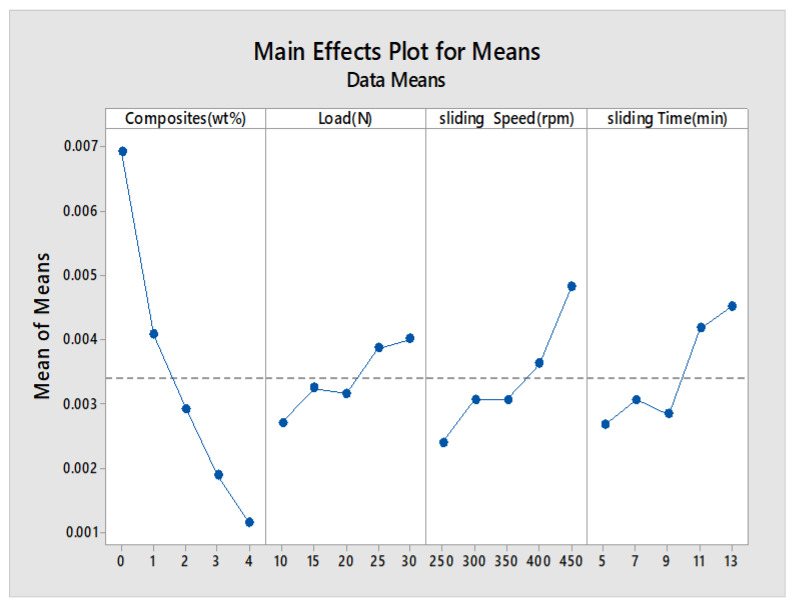
S/N Ratio plot (Weight loss).

The main effects plot and signal to noise ratio are shown in Figure 7, Figure 8, Figure 9 and Figure 10 for wear rate and weight loss, as well as response tables for means and S/N ratio in Table 6, Table 7, Table 8 and Table 9. The smaller condition is chosen as the Taguchi objective, as we need minimal wear rate and weight loss. Figure 7, Figure 8, Figure 9 and Figure 10 state that composite is the most influencing parameter on the output parameter, thereby validating our objective for the addition of reinforcements to enhance the wear behavior of the hybrid metal matrix composites. In the mean of signal to noise ratio represented in Figure 8, from the observation composite, weight increases towards the frictional behavior of Al7075. Load influence is negligible, whereas sliding time and speed partially influence the wear rate. In the aspect of weight loss, which is shown in Figure 9, the effect of composite, load, sliding time, and speed contributed similarly to the wear rate. In Figure 10, the curve trend concerning weight loss indicates the addition of composites for enhancing the wear resistance. With respect to load, wear loss increased proportionally, and by following the same drift, weight loss increased with increment in sliding speed and time.

**Figure 11:**
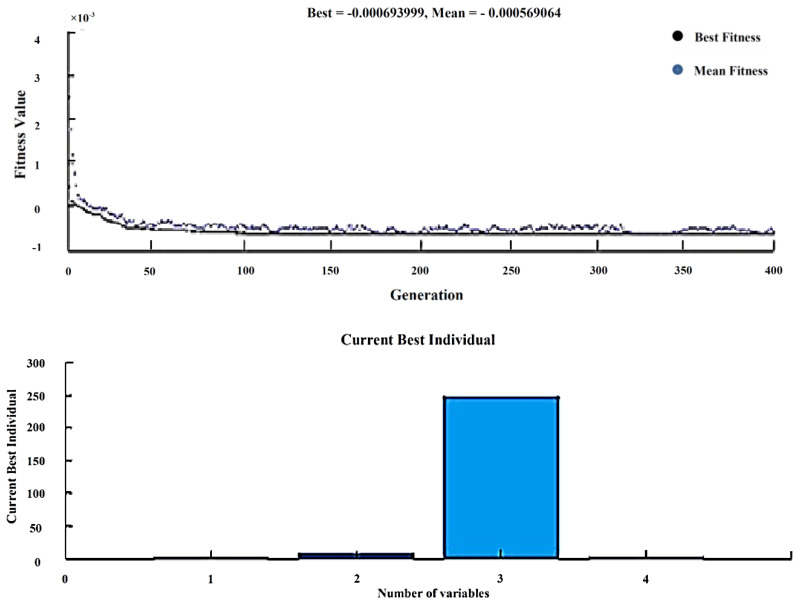
Best and Mean results-Wear rate.

The Genetic Algorithm is one most sophisticated optimization tools implemented with the help of MATLAB. Moreover, the fitness function is generated from the regression equation in Minitab and the number of variables indicating the input process parameters to get the best and mean result of the output response along with corresponding input parameters. The upper and lower bounds of the population are given for better-optimized results. The experimental results were used for finding the wear rate with the help of Taguchi’s orthogonal array, and the regression equation is created in Minitab. The fitness function shown in Equation (1) is derived from regression equations and given as input along with the input process variables to the GA optimization tool, as shown in Figure 11.

(1) $$\begin{array}{cc} Fitness\;function & =@(x)+0.001274-0.001170\cdot x(1)\\ & +0.000038\cdot x(2)+0.000007\cdot x(3)+0.000136\cdot x(4) \end{array}$$

After solving, GA provides the best input as composites (4%), load (10 N), sliding speed (250 rpm), and sliding time (5 min), and the best wear rate as 0.0005955. The experiment is carried with the above input, and the wear rate was found to be 0.000624521, indicating the validation of GA results. Shown in Table 10.

### 4.2. Analysis of Weight Loss

**Figure 12:**
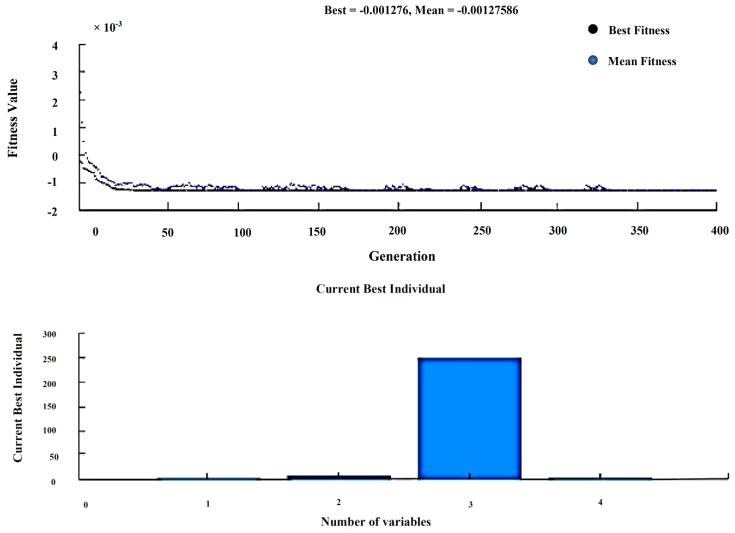
Best and Mean results—Weight loss.

The above procedure was carried out for finding weight loss with the help of Taguchi’s orthogonal array, and the regression equation is created in Minitab. Fitness function shown in Equation (2), is derived from regression equations and given as input along with the input process variables to the GA optimization tool, as shown in Figure 12.

(2) $$\begin{array}{cc} Fitness\;function & =@(x)+0.00016-0.001354\cdot x(1)\\ & +0.000064\cdot x(2)+0.000010\cdot x(3)+0.000168\cdot x(4) \end{array}$$

After solving, GA provides the best input as composites (4%), load (10 N), sliding speed (250 rpm), and sliding time (5 min), and the best weight loss as 0.001276. The experiment is carried with the above input, and the wear rate was found to be 0.0014231, indicating the validation of GA results, as shown in Table 11.

### 4.3. Wire Cut-EDM

**Figure 13:**
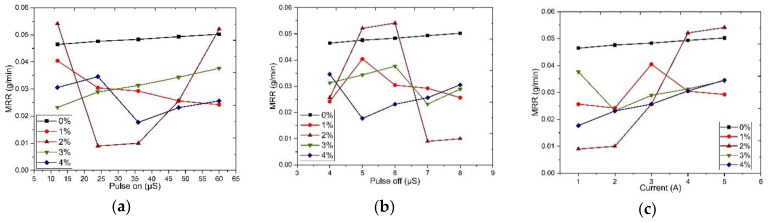
Material removal rate response according to Pulse and Current: (**a**) pulse on; (**b**) pulse off; (**c**) current.

Similar to the wear rate and weight loss Taguchi method, material removal rate was determined through wire EDM, with input parameters such as composites, pulse off, pulse on, and peak current as factors and various weight percentages as level. Minitab, a statistical analysis software, chooses the best L_25_ orthogonal array. Factors such as composites (0%, 1%, 2%, 3%, 4%), pulse on (12, 24, 36, 48, 60) µS, pulse off (4, 5, 6, 7, 8) µS, and current as (1, 2, 3, 4, 5) A are the input parameters, and MRR is the output parameter, as shown in Table 12, and the plotted graph is illustrated in Figure 13.

**Figure 14:**
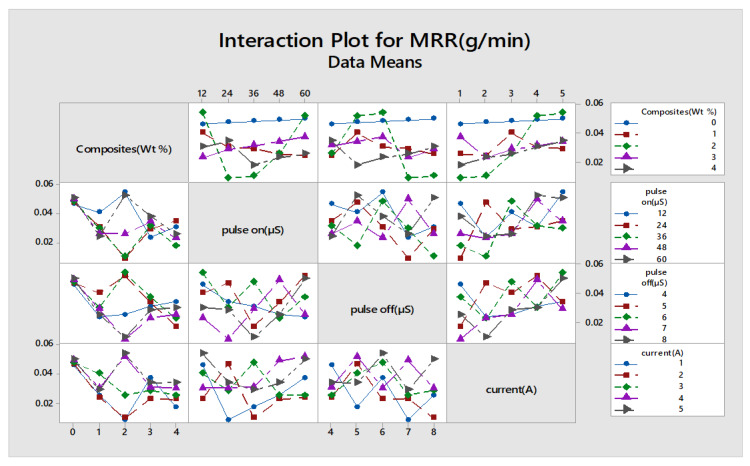
Interaction plot for MRR.

As the same procedure followed for wear rate and weight loss, the percentage of contribution was found for input parameters such as composites, pulse off, pulse on, current to corresponding response parameters such as material removal rate and interaction plot, as shown in Figure 14. Table 13 explains that the higher the addition of reinforcements, the less the material removal rate. A full interaction plot also helps in easy visualization of the impact of the influencing parameters.

**Figure 15:**
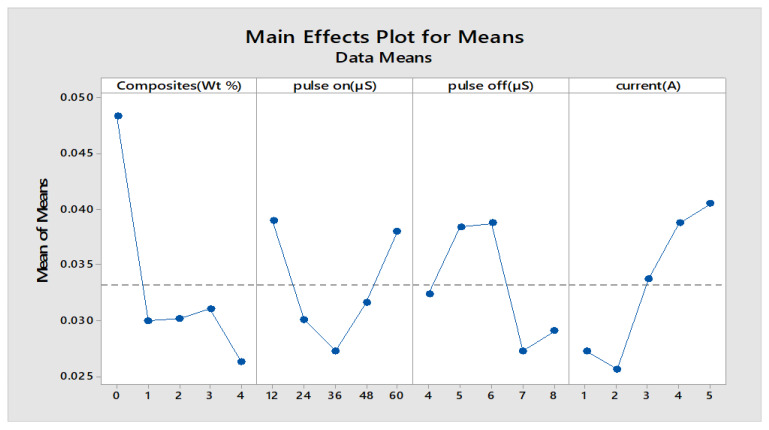
Main effects plot for MRR.

**Figure 16:**
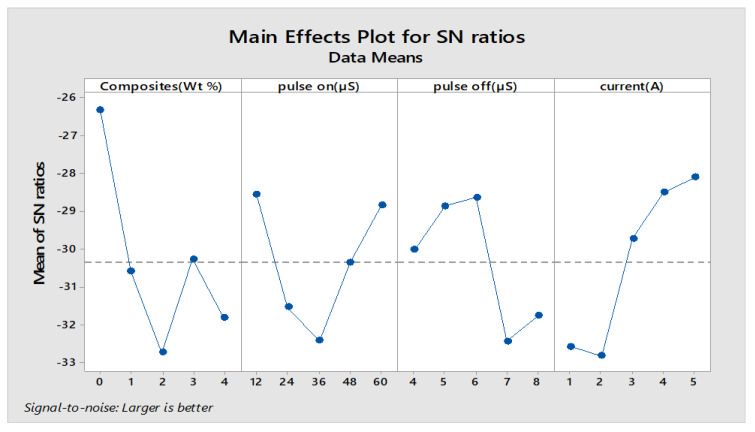
S/N Ratio plot for MRR.

The Minitab statistical software was used to determine the most influencing input factors that affect the responses. Furthermore, this gives a detailed ranking of the influencing parameters. The smaller condition is chosen for wear rate and weight loss, whereas for material removal rate, the better is selected for determining the machining capability of the material. The main effects plot and S/N ratio plot are shown in Figure 15 and Figure 16. The response table for means and S/N are shown in Table 14 and Table 15.

Table 14 infers that the composites are given a higher rank of influencing variables, and this clearly explains the impact of various weight percentages of reinforcements contributing to MRR. The addition of reinforcements reduces the MRR, as it increases the hardness and mechanical strength of that MMC, owing to much less machining capability. The experimental results were deployed for finding MRR with the help of Taguchi’s orthogonal array; the regression equation is created in Minitab. The fitness function shown in Equation (3) is derived from regression equations and given as input along with the input process variables to the GA optimization tool.

(3) $$\begin{array}{cc} Fitness\;function & =@(x)+0.0408-0.00430\cdot x(1)\\ & -0.000004\cdot x(2)-0.00179\cdot x(3)-0.00395\cdot x(4) \end{array}$$

**Figure 17:**
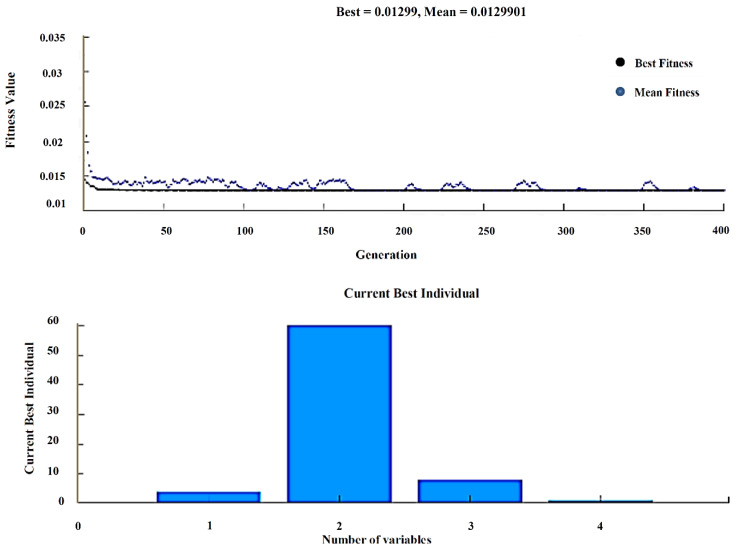
Best and Mean results–MRR.

The GA provides the best input values along with the corresponding MRR best and means values shown in Figure 17. An experiment is carried for that particular input combination, and it is clear that both the GA and experimental results are in closed accuracy, as shown in Table 16.

**Figure 18:**
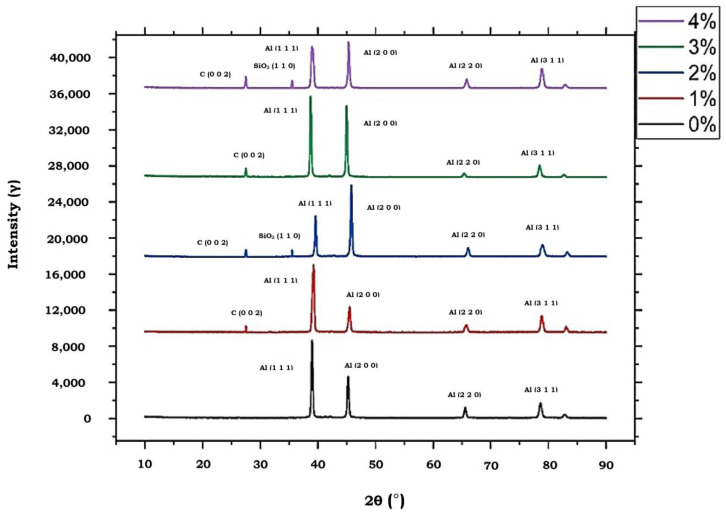
Intensity Vs. Degree in 2 Theta.

Figure 18 gives the X-Ray Diffraction (XRD) results of five different compositions (0, 1, 2, 3, 4) wt.% of AA7075 reinforced with MWCNTs and PFA. The graph shows the different diffraction peaks. The graph denotes that the largest peaks contain the presence of aluminium. Furthermore, the graph shows one major peak and three minor peaks for aluminium. It also indicates the presence of MWCNTs and PFA in small peaks. From the graph, it is proved that with the increase in reinforcement particles wt.%, the intensity of reinforcement particles also gets increased. From the graph, it can also be seen that the peaks of reinforcement particles increase with an increase in wt.%. The work in [29] shares the same XRD peaks on carbon nanotubes as a crystalline form. There is also a similar peak of CNT obtained in the aluminium matrix, in which CNT was incorporated in the aluminium matrix through the induction melting process [30].

## 5. Conclusions

The microstructure images of various wt.% show that the reinforcement particles are dispersed evenly in the base metal matrix. The reinforcement particles are deposited in the grain boundary, which results in the increased strength of the MMC. As the wt.% increases beyond 3%, the agglomeration of reinforcement particles is found. Further, this decreases the strength of the alloy. The composition percentage of reinforcement affects the hardness of the material, and there exists a critical value for the percentage of reinforcement addition that starts deteriorating the synonym of tailored metal matrix composites, due to the accumulation of MWCNTs and PFA over the base matrix. Ultimate tensile strength was highest for 4 wt.%, where the value was 165 MPa. In lower load conditions, abrasive wear was found; meanwhile, for higher load conditions it was adhesive wear. From the Taguchi analysis, it is clear that the most influencing parameters are composites (wt.%) followed by speed (rpm), time (minutes) and load (N), that provides minimum wear rate and weight loss, as it is widely visualized through the main effects plot and signal to noise ratio. The analysis of variance of MRR shows that the percentage of contribution of weight percentage is (38.1%), followed by current (22.61%), pulse on (14.1132%), and pulse off (13.14%), indicating that the weight percentage plays an important role. The analysis of variance of wear rate shows that the percentage of contribution of weight percentage is (82.55%), followed by speed (9.42%), time (4.65%), load (2.34%). The analysis of variance of weight loss shows that the percentage of contribution of weight percentage is (72.54%), followed by speed (10.94%), time (6.566%), load (3.379521%). From the genetic algorithm for MRR, the best input process parameters were found to be composites (4 wt.%), pulse on (60 µS), pulse off (8 µS), and peak current (1 A) for obtaining optimum MRR. Similarly, from a genetic algorithm of wear rate, the best input as composites (4 wt.%), load (10 N), sliding speed (250 rpm), sliding time (5 min), and best wear rate as 0.0005955 is the optimized response. The genetic algorithm for weight loss also gives the best weight loss as 0.001276 for the best-optimized input parameters as composites (4 wt.%) load (10 N), sliding speed (250 rpm), and sliding time (5 min). From XRD analysis, the highest peaks confirmed the presence of aluminum. The presence of MWCNTs and PFA was also witnessed using microstructure images. From the graph, it is inferred that the reinforced particles are evenly dispersed in the base metal matrix. The stratagem of this combination was aimed at fabricating the AA7075 hybrid MMC that is appropriate for aerospace, and an other wide range of applications, based on the satisfying mechanical properties and wear behavior of the synthesized composites. 

## Figures and Tables

**Table 1:** Input parameters of wear rate, weight loss.

Sl.No	Input Parameter	Range
1	Composites (Wt %)	0, 1, 2, 3, 4, 5
2	Load (N)	10, 15, 20, 25, 30
3	Speed (rpm)	250, 300, 340, 400, 450
4	Time (min)	5, 7, 9, 11, 13

**Table 2:** Input parameters of material removal rate.

Sl. No	Input Parameter	Range
1	Composites (Wt %)	0, 1, 2, 3, 4, 5
2	Pulse on (µS)	12, 24, 36, 48, 60
3	Pulse off (µS)	4, 5, 6, 7, 8
4	Current (A)	1, 2, 3, 4, 5

**Table 3:** L_25_ array for weight loss and wear rate.

Experiment	Composites (wt.%)	Load (N)	Sliding Speed (rpm)	Sliding Time (min)	Wear Rate (mm^3^/m)	Weight Loss (g)
1	0	15	300	7	0.0056	0.0056
2	0	10	250	5	0.0044	0.0044
3	0	25	400	11	0.0067	0.0078
4	0	20	350	9	0.0059	0.0059
5	0	30	450	13	0.0083	0.0109
6	1	20	400	13	0.0049	0.0044
7	1	10	300	9	0.0034	0.0029
8	1	30	250	7	0.0038	0.0034
9	1	15	350	11	0.0045	0.0042
10	1	25	450	5	0.0055	0.0055
11	2	15	400	5	0.0030	0.0030
12	2	10	350	13	0.0029	0.0029
13	2	30	300	11	0.0032	0.0030
14	2	20	450	7	0.0038	0.0038
15	2	25	250	9	0.0024	0.0019
16	3	15	450	9	0.0026	0.0022
17	3	30	350	5	0.0014	0.0014
18	3	25	300	13	0.0032	0.0032
19	3	20	250	11	0.0021	0.0011
20	3	10	400	7	0.0017	0.0016
21	4	15	250	13	0.0014	0.0012
22	4	10	450	11	0.0021	0.0017
23	4	20	300	5	0.0009	0.0006
24	4	25	350	7	0.0011	0.0009
25	4	30	400	9	0.0017	0.0013

**Table 4:** Analysis of Variance–Wear Rate.

Analysis of Variance–Wear Rate						
Source	DF	Adj SS	Adj MS	F-Value	*p*-Value	Percentage of contribution
Composites (wt.%)	4	0.000071	0.000018	170.87	0	82.55814
Load (N)	4	0.000002	0.000001	5.67	0.018	2.325581
Sliding Speed (rpm)	4	0.000008	0.000002	18.83	0	9.302326
Sliding Time (min)	4	0.000004	0.000001	10.16	0.003	4.651163
Error	8	0.000001	0 wt.%	-	-	1.162791
Total	24	0.000086	-	-	-	100.0000

**Table 5:** Analysis of Variance–Weight loss.

Analysis of Variance–Weight Loss						
Source	DF	Adj SS	Adj MS	F-Value	*p*-Value	Percentage of contribution
Composites (wt.%)	4	0.0001	0.000025	52.5	0	72.9927
Load (N)	4	0.000006	0.000001	2.91	0.093	4.379562
Sliding speed (rpm)	4	0.000015	0.000004	7.98	0.007	10.94891
Sliding time (min)	4	0.000009	0.000002	4.74	0.03	6.569343
Error	8	0.000004	0	-	-	2.919708
Total	24	0.000137	-	-	-	100.0000

**Table 6:** Response Table for S/N ratio (Wear rate).

Level	Composites (wt.%)	Load (N)	Sliding Speed (rpm)	Sliding Time (min)
1	44.37	51.24	51.71	52.15
2	47.22	50.24	51.02	51.28
3	50.38	50.73	51.70	50.69
4	53.52	50.03	50.18	49.48
5	57.22	50.48	48.09	49.11
Delta	12.85	1.21	3.62	3.05
Rank	1	4	2	3

**Table 7:** Response Table for Means (Wear rate).

Level	Composites (wt.%)	Load (N)	Sliding Speed (rpm)	Sliding Time (min)
1	0.006180	0.002900	0.002820	0.003040
2	0.004420	0.003420	0.003260	0.003200
3	0.003060	0.003520	0.003160	0.003200
4	0.002200	0.003780	0.003600	0.003720
5	0.001440	0.003680	0.004460	0.004140
Delta	0.004740	0.000880	0.001640	0.001100
Rank	1	4	2	3

**Table 8:** Response Table for Means (Weight loss).

Level	Composites (wt.%)	Load (N)	Sliding Speed (rpm)	Sliding Time (min)
1	0.006920	0.002700	0.002400	0.002667
2	0.004080	0.003240	0.003060	0.003060
3	0.002920	0.003160	0.003060	0.002840
4	0.001900	0.003860	0.003620	0.004175
5	0.001140	0.004000	0.004820	0.004520
Delta	0.005780	0.001300	0.002420	0.001853
Rank	1	4	2	3

**Table 9:** Response Table for S/N ratio (Weight loss).

Level	Composites (wt.%)	Load (N)	Speed (rpm)	Time (min)
1	43.63	51.99	53.70	53.91
2	48.00	50.92	52.12	51.93
3	50.90	52.75	52.17	52.13
4	55.04	50.52	50.68	48.89
5	59.38	50.78	48.28	49.09
Delta	15.74	2.23	5.42	5.03
Rank	1	4	2	3

**Table 10:** Comparison of optimized results—Wear rate.

Methods	Composites (wt.%)	Load (N)	Sliding Speed (rpm)	Sliding Time (min)	Wear Rate (mm^3^/m)
GA results	4	10	250	5	0.0005955
Experiment results	4	10	250	5	0.000624521

**Table 11:** Comparison of optimized results—Weight loss.

Methods	Composites (wt.%)	Load (N)	Sliding Speed (rpm)	Sliding Time (min)	Weight Loss (g)
GA results	4	10	250	5	0.001276
Experiment results	4	10	250	5	0.0014231

**Table 12:** L_25_ array for Material Removal Rate.

Exp. No.	Composites (Wt %)	Pulse On (µs)	Pulse Off (µs)	Current (A)	MRR (g/min)
1	0	12	4	1	0.0464300
2	0	24	5	2	0.0476000
3	0	4	6	3	0.0483200
4	0	5	7	4	0.0493200
5	0	6	8	5	0.0502123
6	1	7	5	3	0.0404200
7	1	8	6	4	0.0304210
8	1	36	7	5	0.0292321
9	1	48	8	1	0.0256421
10	1	60	4	2	0.0242123
11	2	12	6	5	0.0541325
12	2	24	7	1	0.0089766
13	2	36	8	2	0.0099765
14	2	48	4	3	0.0256521
15	2	60	5	4	0.0521110
16	3	12	7	2	0.0232123
17	3	24	8	3	0.0288970
18	3	36	4	4	0.0313130
19	3	48	5	5	0.0343210
20	3	60	6	1	0.0376500
21	4	12	8	4	0.0305421
22	4	24	4	5	0.0345600
23	4	36	5	1	0.0176932
24	4	48	6	2	0.0231234
25	4	60	7	3	0.0256422

**Table 13:** Analysis of Variance–MRR.

Analysis of Variance						
Source	DF	Adj SS	Adj MS	F-Value	*p*-Value	Percent of Contribution
Composites(Wt.%)	4	0.001509	0.000377	6.79	0.011	38.65266
Pulse On(µS)	4	0.000513	0.000128	2.31	0.146	13.14037
Pulse Off(µS)	4	0.000554	0.000138	2.49	0.126	14.19057
Current(A)	4	0.000883	0.000221	3.98	0.046	22.61783
Error	8	0.000444	0.000056	-	-	11.37295
Total	24	0.003904	-	-	-	100.0000

**Table 14:** Response Table for Means (MRR).

Level	Composites (wt.%)	Pulse ON (µS)	Pulse OFF (µS)	Current (A)
1	0.04838	0.03895	0.03243	0.02728
2	0.02999	0.03009	0.03843	0.02562
3	0.03017	0.02731	0.03873	0.03379
4	0.03108	0.03161	0.02728	0.03874
5	0.02631	0.03797	0.02905	0.04049
Delta	0.02206	0.01164	0.01145	0.01487
Rank	1	3	4	2

**Table 15:** Response Table for S/N ratio (MRR).

Level	Composites (wt.%)	Pulse ON (µS)	Pulse OFF (µS)	Current (A)
1	−26.31	−28.57	−30.02	−32.59
2	−30.61	−31.55	−28.86	−32.84
3	−32.75	−32.43	−28.64	−29.72
4	−30.27	−30.36	−32.45	−28.51
5	−31.82	−28.85	−31.78	−28.10
Delta	6.44	3.86	3.82	4.74
Rank	1	3	4	2

**Table 16:** Comparison of optimized results—MRR.

Methods	Pulse ON (µs)	Pulse OFF (µs)	Current (A)	MRR (g/min)
GA results	60	8	1	0.01299
Experimental results	60	8	1	0.014686
